# Dogs take into account the actions of a human partner in a cooperative task

**DOI:** 10.1098/rspb.2022.2189

**Published:** 2023-02-22

**Authors:** Mayte Martínez, Lauren M. Robinson, Sarah F. Brosnan, Friederike Range

**Affiliations:** ^1^ Domestication Lab, Konrad Lorenz Institute of Ethology, University of Veterinary Medicine Vienna, Savoyenstraße 1a, A-1160 Vienna, Austria; ^2^ Language Research Center, Georgia State University, Atlanta, GA 30303, USA; ^3^ Departments of Psychology and Philosophy, Neuroscience Institute, Center for Behavioral Neuroscience, Georgia State University, Atlanta, GA 30303, USA

**Keywords:** cooperation, coordination, social behaviour, dog

## Abstract

Humans stand out for their capacity to flexibly cooperate, possibly because they understand their partners' role. Researchers have explored if such understanding is unique to humans by assessing whether non-human species wait to manipulate a cooperative apparatus until a delayed partner arrives. If animals do wait, then it is assumed that they recognize the need for a partner. However, success in these tasks may be the result of social facilitation, while failure may be due to poor inhibitory control. Moreover, this approach does not test if animals take their partners’ actions into account. Here we trained dogs to press a button simultaneously with their human partner. Afterwards, we tested them in several conditions to disentangle which elements of their partner's behaviour they take into account. Dogs waited to press the button until the delayed partner arrived, the button was available to the partner and the partner acted (pressed the button). We found no relationship between inhibitory control and success. We conclude that dogs are not merely reacting to the presence of their human partners, but are also taking their actions into account when coordinating with them.

## 1. Background

Cooperative interactions, defined as two or more individuals working together towards a common goal [1], are widespread in the animal kingdom [2]. The cognitive abilities underlying those behaviours differ across species [3], especially in regard to understanding the role of their partners. While humans recognize the importance of their partner's contributions in a cooperative task, allowing for a unique capacity to flexibly cooperate [4,5], other species may achieve functionally cooperative outcomes as a by-product of individual behaviour, without any understanding of the cooperative situation (i.e. mound-building termites [6]). Finally, some species may only pay attention to a partner being present, while others also take their partner's actions into account. This last aspect is especially important in cooperative interactions that require the subjects to coordinate their actions in time and space, since attending only to the presence or absence of a partner may not be specific enough to succeed [7,8] and it may also allow for free-riding without consequences (e.g. being present but not actively participating in hunting or group defence) [1].

Most studies exploring the extent to which animals understand the role of their cooperative partner rely on the loose-string task [9], in which two individuals must simultaneously pull two ends of a string to bring a platform baited with food within their reach. The string is attached through loops in a way that if only one animal pulls, it comes loose and the food can no longer be obtained. The task can be altered by holding one animal back so that the first one needs to wait for the partner before pulling the string (delay condition). If the subject waits for its partner before pulling, it is thought to understand the need of a partner. A variety of species, from primates [9–11] to other mammals [12,13] and birds [14–16], have been successful in this condition.

Despite its widespread use [17], the delay condition is not exempt from criticisms. For instance, subjects might simply learn to pull the rope when it is tense or moving due to their partner's pulling [8]. Even if this problem can be addressed by replacing the string with handles [18–20], bars [21,22] or buttons [23,24], the delay condition only shows that the subjects are more likely to pull the rope in the presence of a partner, which can be the result of social facilitation [25]. Moreover, failing to wait for a partner does not directly translate into a lack of understanding of the cooperative situation. Instead, waiting behaviour can be restricted by the subject's capacity to inhibit the immediate action of pulling the rope (i.e. inhibitory control [26]). Inhibitory control has been found to be relevant in cooperative contexts, both in humans [27,28] and canids [29,30], but its influence on the successful accomplishment of the delay condition has not been experimentally tested yet.

Given the constraints of the delay condition, researchers have also used other measures as evidence of the animal's understanding of their partner's role, such as how often the subjects gaze at their partners during the task [9,21] and whether the subject recruits a partner when it is needed [9,11,31]. Importantly, all of these studies assess whether animals consider their partner's presence, but further research is needed to evaluate whether animals pay attention to the actions of their partner. Here we investigate whether pet dogs, when cooperating with a human, are only paying attention to the presence of the partner and the apparatus, or also to the actions of their partners.

Dogs are thought to have evolved a genetic predisposition for cooperation with humans [32], making them the ideal subjects for this study. Two studies using the loose-string task showed that dogs waited for the human in the delay condition and even recruited a partner by opening a door giving the partner access to the apparatus [31]. Dogs also succeeded in coordinating with a human partner in a set-up with two pulling apparatuses, by first waiting for and then following them [33]. These results suggest that dogs understand, at minimum, that they need a partner to solve the tasks.

In the present study, we used a button-press task [23] that, unlike the loose-string task, does not provide kinaesthetic feedback (i.e. animals cannot feel the rope's tension and the food is not visible and does not move towards the subjects when they pull [34]). While this prevents the animals from using the apparatus movement as a cue to solve the task, one potential disadvantage is that they cannot spontaneously understand the consequences of their actions. For this reason, as in similar studies [13,15,23,35], we implemented a series of training steps until the animals learned to press the buttons at the same time as their human partners (owners). Then, to investigate which strategy the dogs followed to solve the task, dog–owner dyads were tested in several conditions in which either the partner (delayed-partner (DP) condition), the partner's button (delayed-button (DB) condition) or the moment when the partner pressed the button (delayed-action (DA) condition) was delayed.

If the dogs only pay attention to the apparatus, they can only succeed in the DB condition. If dogs understand that their partners must be present to accomplish the task, they should wait for their partner in the DP condition, but not be successful in other controls. However, this behaviour could also be explained in terms of social facilitation or associative learning between the presence of their partner in front of the button and receiving a reward. To test if the dogs also pay attention to the actions of their partner, we introduced the DA condition, in which dogs can only succeed if paying attention to their partner's pressing behaviour. Finally, establish the chance levels of success and to assess if the dogs pressed the button by following other cues unrelated to the presence or actions of their partners, we designed a non-visibility (NV) control condition, in which the dogs could not see or hear their partner.

To further test for the effect of dogs' inhibitory control in their performance, we measured the inhibitory control of each subject using both a behavioural task and a questionnaire. We predicted that dogs with higher levels of inhibitory control would be less likely to prematurely manipulate the apparatus, and thus more successful in our coordination task.

## 2. Methods

### (a) Subjects

Twenty-one family pet dogs (11 females, mean age ± s.d.: 4.82 ± 3.32 years) of various breeds (see electronic supplementary material for details) were included in the final sample. We tested a similar number of dogs as has been used in previous studies (e.g. [11–16]). The study was conducted at the Clever Dog Lab at the University of Veterinary Medicine Vienna, in an empty test room (7 × 6 m) and always by the same experimenter.

### (b) Inhibitory control measures

Inhibitory control is considered a collection of various processes (e.g. motor inhibition, attentional inhibition and self-regulation) that rarely correlate across tasks [26]. Hence, to capture possible inhibitory control skills of each subject, we considered it important to include more than one measure. Here, we evaluated the dogs’ inhibitory control with two different measures. First, the dog owners filled in the Dog Impulsivity Assessment Scale (DIAS), which is a validated questionnaire about their dog's impulsivity in daily situations [36]. Second, dogs were tested in a motor inhibition task (the box test) in which the subject had to retrieve a piece of sausage located inside a transparent box after learning to retrieve it from an opaque box (for details, see electronic supplementary material, text and figure S1). We followed the same procedure as was used by Brucks *et al*. [26]. The food was only accessible from one side of the box, forcing the dogs to inhibit reaching for the food directly and instead moving around the box to find the open side. We randomly alternated the position of the open side of the box (left, right or back) and the position of the sausage inside the box (centre of the box or deep, touching the wall opposite to the open side). All the combinations (side of the box and centre versus deep) were tested once, in random order, across a total of six trials. Every test trial started with the dog being handled by the owner, who sat 2 m away from the box and was instructed to remain silent. The experimenter first showed a piece of sausage to the dog, then placed a curtain in between the dog and the box so that the dogs could not see the baiting process, and then placed the sausage inside the box. She then removed the curtain, stepped back (1.5 m) and looked at the owner as a signal to release the dog, which was then free to approach and retrieve the food. After they ate the food, or after 30 s, the trial was finished, and the owner called the dog back to the starting position. During the trials, the experimenter remained still and looked at the ground. As a measure of motor inhibition, we coded the frequency of errors across trials (the number of times that the dog touched the surface of the box with the paw or the nose in each trial) [37].

### (c) Coordination task

Dogs were partnered with their owners in the coordination task. Dogs and partners were required to press a button at the same time (within a 2 s time window) to receive a food reward. Dog–partner dyads were together in the room but separated by a wire fence that allowed them to see each other; owners were instructed not to speak to or look at their dog during the test trials. Dogs were always free to move and to decide when to interact with the apparatus. The behaviour of the partner was different depending on the condition (see below).

The apparatus consisted of two red buttons (20 cm of diameter) that the experimenter could slide towards the partner and/or the dog (see electronic supplementary material, text and figures S2 and S3). To prevent the experimenter from influencing the behaviour of the dogs, she was hidden in an experimental chamber (approx. 1.5 m × 1.5 m). The experimenter observed and recorded the dogs and their partners via cameras connected to her laptop. Using a digital clock, she monitored the time between the moment when one member of the dyad pressed the button and when the other member pressed it. Importantly, partners were instructed to press the button only one time; the experimenter considered only the first time the dog pressed. This prevented the dyads from succeeding by chance if the dog repeatedly pressed the button. in the case of success (i.e. the partner and the dog press the button within less than 2 s of each other) a ‘success sound’ (we assigned clicker or ‘marker’ word to each dog, depending on the dog's previous training) was played and then the experimenter delivered the food rewards to the dog (usually one piece of dry food, see electronic supplementary material and table S1) and the partner (peanut) using plastic cylinders attached to the fence. We introduced the ‘success sound’ as a secondary reinforcer to compensate for the delay between the correct response and the delivery of the food. In case of failure (the dog pressed the button too early, too late or did not press at all), a ‘failure sound’ (buzzer) was played, the experimenter pulled the button/s back without delivering any rewards, and after 2 s a new trial began.

All the dogs went through a training phase followed by four different test conditions (see below). We used a within-subjects design in which every dog participated in all the conditions across 4 days (one condition per day) in a counterbalanced order.

#### (i) Training

Dogs completed several training steps in which they learned to wait for 3 s until their partner's button was available and the dyad could press together. As in the test, we used a 2 s window around the human and dog presses to determine simultaneity (for details about the training, see electronic supplementary material). In *step 1*, the dogs were individually trained to press the button in less than 1 s after the experimenter showed it. The criterion was set at success in seven consecutive trials. In *step 2*, the dogs performed the task with the partners who sat in front of the location where their button would be presented. In every trial, the experimenter presented both buttons simultaneously and both, dog and partner, were required to press their respective buttons at the same time. We conducted blocks of 20 trials until the dogs succeeded on at least 14 out of 20 trials in two consecutive blocks.

In *step 3*, we introduced the delay. The experimenter showed the subject's button first and then, after a delay of 3 s, the partner's button. Therefore, the dogs had to wait until both buttons were presented to press the button at the same time as their partner. We conducted blocks of 20 trials until the dogs succeeded 14 out of 20 trials.

In *step 4*, we introduced some changes to minimize extraneous cues. First, partners were instructed to press the button without making any noise and a recording of the sound of the button being pressed was played continuously during all the trials to reduce the effect of the sound as a cue. Additionally, to control for any facial/gaze cue, partners were asked to wear sunglasses and a short curtain was placed between them and the dogs in a way that the dogs could monitor the hand movements of their partners, but they could not see their faces. We included both elements (curtain and sunglasses) in case the curtain would move during the test. We conducted blocks of 20 trials until the dogs succeeded 14 out of 20 trials in two consecutive blocks.

Dogs needed an average of 2.3 blocks (range 2–4) to pass the *step 2* of the training, an average of 15.6 blocks (range 6–25) to pass *step 3* and an average of 5.4 (range 2–21) to pass *step 4* (see electronic supplementary material, table S1).

#### (ii) Test

**Figure 1.**
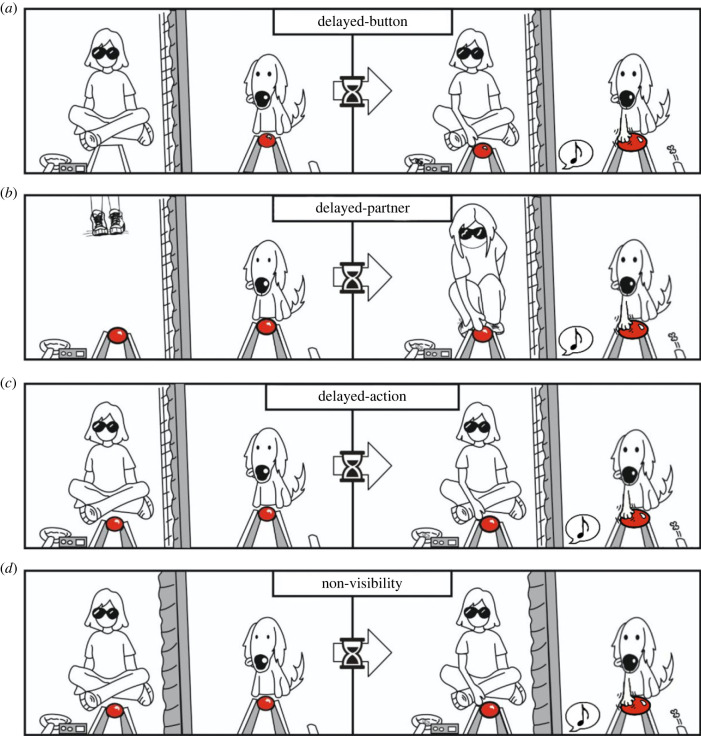
Test conditions. (*a*) Delayed-button. (*b*) Delayed-partner. (*c*) Delayed-action. (*d*) Non-visibility. For each condition, the picture on the left shows the position of the partner and the button at the beginning of the trial. After the delay (represented by the arrow and the sand-clock), the picture on the right shows the successful completion of the trial. Button on the right panels was drawn bigger only to highlight the pressing action. Note the extension of the curtain in the non-visibility condition (*d*).

Dogs were tested in four test conditions (figure 1) that complemented each other to explore which strategy dogs followed to succeed in the task:

*DB condition*. This test followed the same procedure as the training trials except that after presenting the dog's button, the experimenter waited for the delay period before presenting the partner's button. To succeed, dogs had to wait until both buttons were visible (figure 1*a*). This procedure was identical to the training, except for the length of the delay period. While in the training this delay was constant (3 s), in the test condition dogs experienced variable delays ranging from 3 to 9 s (see below).

*DP condition*. Every trial started with the partner standing up at the back of the room. Then, the experimenter showed both buttons at the same time and said the number of seconds that the partner had to wait before approaching the apparatus. The partner could count those seconds using a digital clock attached to the wall. After that time, if the dog was still waiting without having pressed the button, the partner walked towards the apparatus and pressed the button. Success in this condition required that dogs pay attention to the presence of their partner, and only press the button once their partner is close to the button (figure 1*b*).

*DA condition*. During this condition, the partner sat in front of the location where the button would be presented, as in the training and the DB condition. The experimenter presented the two buttons at the same time and said the number of seconds that the partner had to wait before pressing the button. The partner could count those seconds using a digital clock, this time attached to the apparatus. After the delay, if the dog was still waiting, the partner pressed the button. While in the DB and DP condition, it was not necessary to pay attention to the specific actions of the partner; the DA addressed whether subjects coordinated their pressing with their partner's pressing (figure 1*c*).

*NV condition*. To estimate the number of trials the dyads could succeed in by chance, we repeated the procedure of the DA condition adding an opaque curtain between the dog and the partner (figure 1*d*).

Every test condition was divided in two phases. The first phase, the fixed-delay phase, consisted of 10 trials where the delay was always 3 s long. This phase gave us information on how the dogs initially reacted to the changes in the partner's behaviour that were introduced in the test procedure. However, dogs could have succeeded in the fixed-delay phase by becoming accustomed to the 3 s delay. To address this, we added a second phase, the variable-delay phase, to test whether dogs would wait for their partners for different amounts of time. The variable-delay phase consisted of 84 trials with variable delays, as long or longer than in the training phase (3, 6 or 9 s), presented in a pseudo-randomized order (no delay length was ever presented more than three times in a row). Dogs had a 2 min break every 21 trials to drink water and interact with their owners to avoid fatigue. The set-up remained the same as in *step 4* of the training (i.e. partners pressed the button silently, wore sunglasses and a short curtain prevented them from looking at the dogs).

### (d) Coding and analysis

In the coordination task, both for fixed-delay and variable-delay phases, we coded the dogs' and partners’ latencies to press the button from the beginning of the trial (when the first button was shown). With these data, we obtained several measures: success (a trial was considered successful if the difference between the partner's and subject's latency was less than 2; otherwise, we coded it as a failure), type of error (dog pressing the button too early, too late or not pressing), and first member of the dyad to press the button (dog or owner). Additionally, we coded the proportion of time that dogs had their heads oriented towards their partners immediately before pressing the button (see details in the electronic supplementary material).

Using a GLMM, we analysed whether success was influenced by condition (DB, DP, DA or NV). We did this separately for the fixed-delay and the variable-delay phases (fixed-delay and variable-delay model). We also included fixed effects of condition order (if dogs participated in each condition as their first, second, third or fourth test), trial number, subject's age (to account for the high variability in our sample) and, only for the variable-delay model, the length of the delay.

We ran another model to explore whether the proportion of time that dogs spent with the head oriented towards their partners (as a measurement of gaze) was higher in successful trials and whether trial, condition or condition order affected gazing patterns (head orientation model). For this analysis, we included only the DB, DP and DA condition, as in the NV it was not possible to look at the partner.

We further investigated whether dogs just reacted to their partners' actions or whether they anticipated their partners’ movements and pressed their buttons before their partners. We used binomial tests to compare the proportion of trials in which each subject pressed the button before their partner (only successful test trials of the DB, DP and DA conditions) with a probability of 0.5. We ran an additional GLMM to test whether factors like condition, trial number or age affected the probability of the subjects pressing first, as well as how variables those effects were among individuals (first-to-press model).

Finally, we tested whether inhibitory control had an effect in the proportion of trials in which the subjects failed to wait before pressing the button. First, to assess if our two inhibition measures capture distinct aspects of inhibition, we transformed the two measures to the same scale (z-transformation) to test whether they correlated with each other using Spearman correlations. One we proved that the two measures were not correlated, we fitted a GLMM (inhibitory control model) using both of our inhibitory control variables (DIAS questionnaire and box test) as predictors. As a response variable, we used a two-column matrix with the number of failed trials (only in the variable-delay phase of the DB, DP and DA condition) in which the subject pressed too soon in the first column and, to control for the total number of failed trials, a second column with the number of failed trials due to other reasons. As further fixed effects we included the length of the delay, subjects' age and condition. We decided to include only the variable-delay phase as we were interested in the effect of the length of the delay (that was invariable in the fixed-delay phase) and to limit the effect of other variables such as arousal, perseverance or flexibility, that could arguably have a higher effect in the first trials of each testing session (fixed-delay trials).

In each of the GLMMs, we included the theoretically relevant interactions between predictors and fixed effects, subject ID as a random factor and all the identifiable random slopes. To avoid multiple testing [38], we tested the significance of the models with all the fixed effects (full model) compared to models lacking the variables of interest but otherwise identical to their respective full models (see electronic supplementary material, text and table S2 for a detailed description of the models’ construction).

## 3. Results

**Figure 2.**
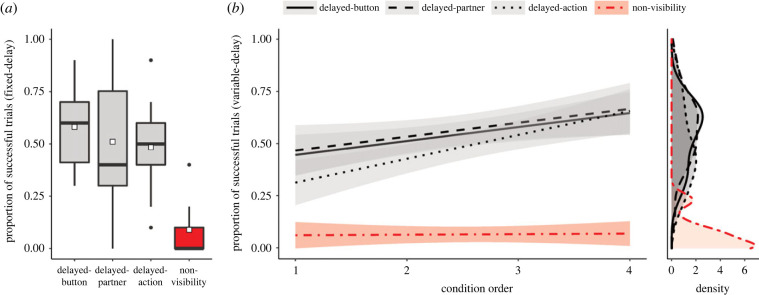
The proportion of success in the coordination task in each condition for the fixed-delay phase (*a*) and variable-delay phase (*b*). The three conditions in which dogs had visual access to their partners are represented in grey, and the control condition in which dogs could not hear or see their partners is represented in red. In (*a*), boxes show the interquartile range and the whiskers show the range of data points from 1.5 times the interquartile range from the upper and lower hinge. Black points represent outliers. Black horizontal bars represent the median and the mean is displayed by a white squared shape. Graph (*b*) shows the regression line and the 95% confidence intervals (shaded area) estimated for a linear model in each condition depending on the condition order. A density plot of the data by condition is displayed to the right.

In the fixed-delay phase, dogs succeeded in about half of the trials in the DB (*M* = 0.566, s.d. = 0.129), DP (*M* = 0.51, s.d. = 0.290) and DA conditions (*M* = 0.484, s.d. = 0.203), while their success drastically dropped in the NV condition (*M* = 0.089, s.d. = 0.129). The fixed-delay model (electronic supplementary material, table S4) revealed that condition influenced the success (*χ*^2^ = 34.300, d.f. = 3, *p* < 0.001), with *post hoc* comparisons indicating that dogs had higher rates of success in the DB (*estimate ±* s.e. = 3.562 ± 0.566, *p* < 0.001), DP (*estimate ±* s.e. = 3.126 ± 0.528, *p* < 0.001) and DA (*estimate ±* s.e. = 2.825 ± 0.525, *p* < 0.001) conditions compared to NV (figure 2*a*). Additionally, dogs improved their performance with more testing experience with different conditions (condition order's *estimate ±* s.e. = 0.564 ± 0.165, *χ*^2^ = 11.128, *p* < 0.05).

One question is whether dogs, independently of whether they succeed, tried to participate in the task when first confronted with each condition. If dogs understood that they need to coordinate with their partner, they would not try to press the button in the NV condition, as their partner is not visible. Looking at the dogs' behaviour in the first trial of the fixed-delay phase, we found that when dogs could see their partners, 16 dogs (84.2%) tried to coordinate by pressing the button in the DB and DP conditions, and 17 (89.5%) in the DA condition. Conversely, in the NV condition, only eight dogs (42%) pressed the button at all (e.g. they refrained from even trying to coordinate if they could not see their partner).

Similarly to the fixed-delay phase, dogs succeeded in the variable-delay phase in approximately half of the trials in the DB (*M* = 0.541, s.d. = 0.151), DP (*M* = 0.565, s.d. = 0.172) and DA (*M* = 0.487, s.d. = 0.186) conditions, while their performance was very poor when they did not have visual access to their partners in the NV condition (*M* = 0.065, s.d. = 0.079). The variable-delay model (electronic supplementary material, table S5) showed that condition influenced success (full–null model comparison likelihood ratio test: *χ*^2^ = 163.8496, d.f. = 3, *p* < 0.001) and that the effect of condition order was different depending on the condition (interaction between condition and condition order: *χ*^2^ = 11.80, d.f. = 3, *p* < 0.008). Specifically, dogs that experienced the DA condition first were worse in that condition than the dogs that started with other conditions (*estimate* = 0.971, *CI* = 0.576–1.365) (figure 2*b*). By contrast, condition order did not have robust effect in the dogs’ performance for any of the other conditions (all the confidence intervals include 0; DB: *estimate* = 0.276, *CI*: −0.138 to 0.690; DP: *estimate* = 0.325, *CI*: −0.089 to 0.739; NV: *estimate* = −0.007, *CI*: −0.368 to 0.355).

There was some learning across trials (trial number *estimate ±* s.e. = 0.072 ± 0.120, *χ*^2^ = 19.99, d.f. = 1, *p* < 0.001) (electronic supplementary material, figure S4), but the wide confidence intervals and the results of the model stability (see electronic supplementary material, table S5) indicate that this effect was not very robust. The model also showed a negative effect of age, with older dogs being less likely to succeed than younger ones (*estimate ±* s.e. = −0.290 ± 0.096, *χ*^2^ = 7.71, d.f. = 1, *p* < 0.05). The length of the delay also had a detrimental effect on success (*estimate ±* s.e. = −0.490 ± 0.051, *χ*^2^ = 35.61, d.f. = 1, *p* < 0.001) (electronic supplementary material, figure S5).

Considering gaze patterns, dogs oriented their heads towards their partner's side of the fence before pressing the button in the majority of trials (see electronic supplementary material, table S6). In the head orientation model (see electronic supplementary material, table S7), we found that dogs looked longer in successful than in unsuccessful trials (effect of success: *estimate ±* s.e. *=* 0.508 *±* 0.084, *χ*^2^ = 20.952, d.f. = 1, *p* < 0.001). Condition was also significant (*χ*^2^ = 17.968, d.f. = 2, *p* < 0.001), with *post hoc* comparisons revealing that they looked longer towards their partners in the DP (*estimate ±* s.e. *=* 0.530 *±* 0.109, *p* < 0.001) and the DA condition (*estimate ±* s.e. *=* 0.263 *±* 0.112, *p* = 0.048) as compared to the DB condition (see electronic supplementary material, figure S6). Moreover, looking increased over trials (condition order: *estimate ±* s.e. *=* 0.187 *±* 0.066, *χ*^2^ = 6.925, d.f. = 1, *p* = 0.008; trial number: *estimate ±* s.e. *=* 0.060 *±* 0.029, *χ*^2^ = 3.840, d.f. = 1, *p* = 0.05). In the trials in which dogs never pressed the button, they were not oriented towards their partners in approximately half of the trials (DB: *M* = 0.57, s.d. = 0.34; DP: *M* = 0.47, s.d. = 0.34; DA: *M* = 0.56, s.d. = 0.29).

In the first-to-press model (see electronic supplementary material, tables S8–S10 and figure S7), there was no effect of the tested variables (condition, trial number or subject's age) in the dogs ‘tendency to press the button before their partners (full–null model comparison: *χ*^2^ = 4.524, d.f. = 4, *p* = 0.340), and most of the dogs (*n* = 15) were more likely to press after their partner pressed.

In the inhibitory control model (see electronic supplementary material, table S11), neither the DIAS's score nor the number of errors in the box test had an effect on whether dogs waited for their partner before pressing the button (full–null model comparison: *χ*^2^ = 14.644, d.f. = 12, *p* = 0.26). Spearman's rank correlation revealed no correlation between the DIAS's score and the number of errors in the box test (*r*_18_ = −0.090, *p* = 0.704).

## 4. Discussion

Although cooperative outcomes are widespread across the animal kingdom, surprisingly little is known about what animals actually understand about their role and that of their partner. In this study, we demonstrated that dogs can coordinate with their owners in a cooperative task, and they did so by paying attention to the actions of their partners.

Because our task lacked any visible mechanisms that could allow the dogs to figure out which actions they were required to perform, we had to train them to ensure they understood the apparatus. This prevents any conclusion about spontaneous cooperation but allowed us to tease apart what strategies dogs may have been using to solve the task and how they can generalize these strategies to new contexts.

Overall, we found that dogs performed much better in the DB, DP and DA condition compared with the NV condition, suggesting that they animals paid attention to the need for the button, the partner and the partner to act on the button, rather than just hitting at random. Similar results were found with a coordinated bar-pulling task in capuchin monkeys [21], including that the monkeys’ success rate decreased when visual access to their partners was blocked. Thus, these studies suggest that dogs (and capuchin monkeys) understand something about the actions of their partners when coordinating on these tasks. Below, we consider these findings in more detail.

While dogs in our study could have solved the DB and DP conditions by following simple rules, such as ‘press the button’ or ‘press when next to a partner’ [8] (see similar results in other species [11–16,23] and dogs paired with humans [31,33]; but see [39]), the DA condition demanded that they pay close attention to the action of the partner. This behaviour could be the result of simple associative learning acquired during the training procedure; however, this was the first time that the dogs had ever experienced their partner delaying her pressing. In this regard, we also found that dogs succeeded in the first trials of the DA (fixed-delay phase), yet many dogs quit pushing from the very first trial in the NV condition, which showed that they did not need extensive experience with every condition to understand and solve the task.

Moreover, the dogs also appeared to pay close attention to their partner's actions, as they were more successful the longer they looked at their partner. Glancing at the partner in a cooperative task has been previously interpreted as evidence of monitoring the partner's behaviour [9,18,19,21] (but see [40]). Considering all together, these findings suggest that dogs learned to pay attention to their partner's presence and actions, which allowed them to adjust their own actions accordingly.

It still remains uncertain whether dogs paid attention to the apparatus or relied only on the movement of their partners. We were not able to address this question as we did not incorporate any condition where partners performed the pressing movement without the button being present. However, if dogs were paying attention only to the movements of their owner and not to the apparatus, one could expect an equal performance among the DB, DP and displayed-pressing conditions, as all of them can be solved by following the partner's hand movement. Conversely, dogs in our study performed better in the DA condition when they had experienced the other conditions before, suggesting that this condition was more difficult, presumably because it requires attention to the partner's actions rather than the apparatus (i.e. the button being shown in the DB condition) or the partner's presence (i.e. partner approaching in the DP condition). This might suggest either that they learned the contingencies of the task, or that when available, they use simpler cues (such as partner presence) to coordinate, and resort to more specific ones only when those simple cues are no longer meaningful. It would be interesting for future work to attempt to disentangle these possible explanations.

One factor that might have affected dogs' success in our task is that they participated together with a human partner. Literature suggests that dogs are attentive to human actions; dogs are faster at finding the solution to a problem when they have previously observed a human demonstrator [41–43] actively seek human contact [44,45] and recognize when they need to recruit a partner [31]. Our study goes beyond previous literature and shows that dogs can actively coordinate their actions with a human in a cooperative situation. However, considering that dogs are known for their cooperation with humans, one may have expected a higher rate of success that the one we found. Indeed, dogs in our study waited for their partners to press the button in approximately half of the trials, in contrast with higher success rates shown in similar tasks by pack-living dogs [31] and other species [13,15,23]. Although a direct comparison among studies is complicated due to methodological differences, one possible explanation is that spontaneous dog–human interactions usually include visual/verbal communication [46]. We, however, instructed human partners to not look or interact with the dog during the test trials so they could not inadvertently cue the dogs, removing that cue and potentially increasing the difficulty for the dogs. It is also interesting that human partners in the present study were the dogs’ owners, and some evidence suggests that dogs pay closer attention to the actions of humans with whom they share a strong bond [47] (but see [48] for a lack of relationship between affiliation and over-imitation), would rescue their owner from a box more often if they express distress [49,50], and exhibit locomotor synchrony with them [51,52]. We are currently exploring whether the relationship between partners and dogs affects their level of coordination in a cooperative task, and hope that further studies will explore whether the dog’ performance is related to the dog–owner interaction style [53]. Of course, some factors were conducive to helping dogs succeed. For instance, dogs were less likely to succeed in trials with longer delays. This is not surprising given that dogs were only trained with a delay of 3 s, in contrast with studies which used a wider range of delays during the training [13,15,23,35,54]. Indeed, most dogs have been shown to have difficulties waiting for more than 2–3 s [26,55], which could be due to a lack in inhibitory control. However, we did not find any effect of individual inhibitory control measures (DIAS score or number of errors in the box test) on the dogs' ability to refrain from pressing the button. This lack of relationship between inhibitory control and animals’ performance in cooperative tasks has also been found in wolves [30] and chimpanzees [56]. Another possibility is that, given that inhibitory control measures are task and context-dependent [26,57–59], we failed to find an effect because the task that we measured aspects of inhibitory control that were not relevant for our cooperative task. Younger dogs also outperformed older dogs, which is similar to other studies that have found that older dogs have reduced cognitive flexibility [60], and are more distractible [61], and less sociable [62], which might have affected their sensitivity to their partner's actions [63].

It is also interesting to reflect on what helped the dogs succeed. A potential explanation for our results is that dogs were using a leader–follower strategy, in which they did what their partner did until they could not see the partner pushing the button. This would be in line with previous research showing that, when coordinating with a human, dogs tend to follow them [33]. Nonetheless, in our study not all the dogs always waited for the partner to press the button. Some of them, perhaps because of the extensive training procedure that we chose to conduct, seemed to be able to anticipate when the partner was going to act, as evidenced by the fact that they tended to press the button just before their partner. Dogs also showed some improvement over the course of the test trials, which could reflect either learning or changes in the dogs' emotional state that supported good problem solving [64,65]. At the beginning of every testing day, dogs were usually very excited, which might have limited their ability to refrain from pressing the button and wait for their partner.

Taken together, our results show that dogs have at least some understanding of the importance of their partner's actions in a cooperative task. Indeed, an enduring question in the literature is the degree to which other animals understand cooperation, and their partner's roles in it. Results such as these suggest that for pet dogs, at least, there is a good understanding of both the contingencies of cooperation and how the partner is involved. While some data suggest that the same is true for other highly cooperative species, such as dolphins [23], capuchin monkeys [21] or chimpanzees [11], it is important to note that our conclusions involve pet dogs working together with their owner. Thus, an important future direction is to shed light on how domestication and life experience affected cooperative skills in dogs, which would require further investigating whether our results generalize to different populations of dogs as well as cooperation with unfamiliar humans or conspecifics. Some research has already been conducted regarding dogs' abilities to coordinate with a conspecific partner in a problem-solving situation [12,39,54,66] but results are mixed, and we are still far from reaching a clear conclusion on whether dogs understand the importance of their conspecific partners in solving a cooperative task. Another key direction will be determining if domestication is the only path to this cooperation, or whether other species also do so, having evolved the ability through different selective pressures. Carefully controlled studies such as these that rule out as many alternative explanations as possible will be key to determining what it is that animals actually understand about cooperation and their role in it.

## Data Availability

All datasets used for the analyses are available as electronic supplementary material [67]. The data are provided in the electronic supplementary material [68].
